# Glomerular filtration rate (GFR) is accurately and precisely measured by a continuous low dose iohexol infusion (CILDI) during acute kidney injury (AKI)

**DOI:** 10.1186/2197-425X-3-S1-A460

**Published:** 2015-10-01

**Authors:** JJ Dixon, K Lane, RN Dalton, IAM MacPhee, BJ Philips

**Affiliations:** St. George's Hospital, General Intensive Care, London, United Kingdom; King's College London, Paediatric Biochemistry, London, United Kingdom; St. George's Hospital, Renal and Transplantation Medicine, London, United Kingdom

## Introduction

AKI is currently defined by changes in Creatinine and urine output, but both parameters have limitations in critically ill patients. We have previously validated a method of measuring GFR using a continuous infusion of low dose Iohexol (CILDI) in patients with stable renal function and calculate that variations >10.3% depicts AKI (p = 0.003). All subjects reached steady state within 10h. In this study we measure the performance of CILDI in patients with different risks of AKI.

AIM. Validate CILDI as a measure of GFR during AKI.

## Methods

11 patients post nephrectomy [NEPH; predictable onset, predicted 50% drop in GFR], 11 patients post vascular surgery [VASC; predicted onset of AKI, unpredictable outcome], and 13 patients with established AKI [AKI; unpredictable onset and outcome] were recruited. CILDI was applied at 0.5mL/h for 24-84h. Urine and serum Iohexol concentrations were measured by tandem mass spectrometry. Accuracy of CILDI was determined by comparing the mean GFR drop in the NEPH group with the predicted 50% change, and by comparing plasma clearance (PC) with renal clearance (RC). Precision was confirmed by measuring the co-efficient of variation (CV) of repeated measurements taken in 2h periods.

## Results

Mean APACHE score was 11 ± 4 (NEPH), 13 ± 2 (VASC) and 18 ± 5 (AKI; p = 0.0004). There was no difference in baseline eGFR (p = 0.70). Mean GFR at 10-14h was 51.4 ± 18.3% of baseline (NEPH), 52 ± 29% baseline (VASC) and 44 ± 31% (AKI group). There was no difference between the predicted drop of 50% and measured drop in the NEPH group (p = 0.81), confirming accuracy of CILDI. Correlation between PC and RC was good: 0.73 (NEPH), 0.82 (VASC) and 0.92 (AKI). The diagnosis of AKI would have been missed in 5 NEPH patients and 4 VASC patients using conventional criteria. In addition, 2 patients who had AKI defined by creatinine, did not have AKI when measured by CILDI. CV was 3.4% overall and small in each group: 3.6% (NEPH), 2.5% (VASC) and 2.1% (AKI).

## Conclusions

CILDI appears to be accurate and precise in measuring changing GFR in patients with AKI. We hypothesise that if our findings are externally validated, then new definition of AKI should incorporate changes in GFR measured by CILDI. CILDI is now ready to be used in studies measuring the effects of AKI.

## Funding

Intensive Care Foundation; St. George's Hospital Charity.

